# Editorial: Neuroimaging in psychiatry 2023: mood disorders

**DOI:** 10.3389/fpsyt.2025.1571482

**Published:** 2025-09-26

**Authors:** Massimo Tusconi, Serdar M. Dursun

**Affiliations:** ^1^ Department of Medical Sciences and Public Health, University of Cagliari, Cagliari, Italy; ^2^ Neurochemical Research Unit, Department of Psychiatry, University of Alberta, Edmonton, AB, Canada

**Keywords:** neuroimaging, biomarkers, neurobiology, mood, bipolar disorder, Bd

The field of neuroimaging has made significant progress in recent decades, significantly influencing the understanding and treatment of psychiatric disorders, particularly mood disorders such as major depressive disorder (MDD) and bipolar disorder (BD) (1–4). These complex conditions, characterized by pervasive mood, affect, and behavioral symptoms, have long been a challenge for diagnosis and treatment because of their multifactorial etiology and the absence of definitive biomarkers (5). The role of neuroimaging in psychiatry has expanded beyond traditional structural imaging techniques to encompass a variety of advanced modalities, including functional magnetic resonance imaging (fMRI), positron emission tomography (PET), diffusion tensor imaging (DTI), and magnetic resonance spectroscopy (MRS) (6–8). These techniques have facilitated a more nuanced understanding of the neural circuits implicated in mood disorders, elucidating abnormalities in brain regions such as the prefrontal cortex, amygdala, hippocampus, and anterior cingulate cortex (9). Neuroimaging has emerged as a crucial tool for unraveling the neurobiological underpinnings of these disorders, providing insights that are beginning to bridge the gap between clinical symptomatology and the basis of the disorders (10–14). Functional studies on Major Depressive Disorderhave identified hypoactivation in the dorsolateral prefrontal cortex (DLPFC) during tasks requiring executive function and cognitive control, as well as hyperactivation of the amygdala in response to negative emotional stimuli (15). These findings have been interpreted as reflecting a dysregulated neural circuit, wherein diminished top-down control from the prefrontal cortex fails to modulate hyperactive limbic structures, leading to the emotional and cognitive disturbances characteristic of depression (16–20). Studies on MDD have documented structural abnormalities such as reduced gray matter volume in the prefrontal cortex and temporal lobes, as well as functional alterations in the ventral prefrontal cortex and striatum (21, 22). Bipolar disorder, with its alternating episodes of mania and depression, presents a more complex neuroimaging profile (12, 23). The Research Topic brings together a diverse array of manuscripts that utilize neuroimaging to explore structural and functional alterations in mood disorders. A central theme emerging across the contributions is the identification of potential neurobiological biomarkers through advanced imaging techniques, with a particular focus on distinguishing features of MDD and BD (24–27). The Research Topic describes multiple aspects of neuroimaging, in particular diagnostic groups (MDD vs. BD), neuroimaging techniques (fMRI, EEG), and insights into targeted brain areas.

In the context of diagnostic stratification, Schreiber et al. identified a significant enlargement of the left vagus nerve cross-sectional area (VN-CSA) in individuals with Major Depressive Disorder (MDD), particularly among those with recurrent episodes. This morphological alteration of the cervical vagus nerve may serve as a novel imaging biomarker, offering potential insights into the somatic underpinnings of depressive pathology.

Additionally, Estudillo-Guerra et al. revealed in their study a trend indicating a higher perfusion imbalance in the left superior and middle frontal gyrus during mania and the right superior and middle frontal gyrus during euthymia phases in participants with Bipolar Disorder Type I.

In the topic concerning neuroimaging techniques (fMRI, EEG), Huang et al. compared functional and structural MRI abnormalities between bipolar and unipolar depression. They found that the BD group exhibited an increased fractional amplitude of low-frequency fluctuation (fALFF) in the hippocampus compared with both the healthy control (HC) and MDD groups.

Furthermore, Liu et al. proposed a multi-scale spatial–temporal local sequential and global parallel convolutional model. This method aimed to improve the diagnostic accuracy of Generalized Anxiety Disorder, particularly in the context of mood instability, using high-frequency electroencephalogram (EEG) signals.

In their investigation of Major Depressive Disorder (MDD) using functional magnetic resonance imaging (fMRI), Endo et al. identified specific dynamic brain activity patterns, referred to as dynamic modes, that occurred with either increased or decreased frequency in individuals with MDD compared to healthy controls. These alterations suggest a disruption in the temporal organization of neural networks, potentially reflecting impaired flexibility and adaptability in brain function (28–30). Such findings contribute to the growing body of evidence supporting the role of dynamic functional connectivity as a potential state-sensitive biomarker in mood disorders.

To complement the theme, Willinger et al. reported weakened effective connectivity between the salience network and the default mode network during the resting state in participants with adolescent depression. They suggested that this pattern may reflect a hierarchical imbalance between the default mode network (DMN) and the salience network (SN).

Chen et al. using a machine learning approach, examined abnormal voxel-mirrored homotopic connectivity in participants with first-episode MDD. They found reduced functional connectivity in the bilateral middle frontal gyrus, fusiform gyrus, medial superior frontal gyrus, and precentral gyrus. These alterations may be linked to depressive symptoms and could serve as a potential biomarker of MDD.

Delving deeper into the discussion regarding the morphobiological aspects of targeted brain areas, Liu et al. observed altered functional activity in the right fusiform gyrus and the left superior temporal gyrus in individuals with treatment-resistant depression following a dual-target accelerated transcranial magnetic stimulation protocol.

Examining structural alterations in participants with MDD, Wang et al. reported a region-specific reduction in cortical thickness, particularly within the left rostral middle frontal gyrus. This thinning exhibited a significant negative correlation with illness duration, suggesting a progressive neuroanatomical deterioration associated with the chronicity of depressive episodes. These findings underscore the potential of cortical metrics, such as reductions in the rostral middle frontal gyrus, as longitudinal markers of disease burden and progression in MDD.

Kijima et al. explored how fronto-striato network function is reduced in participants with MDD highlighting that the reward system network may be an important biological marker of MDD, although careful consideration should be given to age and its association with the severity of the disorder.

Finally, Cong et al. examined hippocampal subfield morphology in participants with first-episode BD type II and major depressive disorder within a drug-naïve Chinese cohort. They reported a significant increase in hippocampal volume, particularly on the left side, observed only in the MDD group compared with healthy controls, and not in the BD-II group. This finding was specific to the studied sample and requires replication in larger, independent cohorts to confirm its validity.

Across the included studies, several key themes emerge, including the potential of specific brain regions (DLPFC, hippocampus) and connectivity patterns (DMN-SN interaction) as diagnostic and therapeutic biomarkers, the utility of machine learning in neuroimaging classification tasks, and the convergence of structural and functional findings in delineating mood disorder subtypes In conclusion, the most recent advances in neuroimaging technology over the past two decades have greatly deepened our understanding of the neurobiological basis of mood disorders (31–34). By revealing the structural and functional abnormalities associated with MDD and BD, these tools have not only improved diagnostic accuracy but also opened new avenues for the creation of personalized medicine strategies. The integration of neuroimaging with other emerging fields, such as genomics, epigenetics, and machine learning, promises a more nuanced approach to psychiatry, in which treatment can be tailored to the individual’s unique neural and genetic profile. By continuing to harness the power of neuroimaging, the convergence of technology and neuroscience holds promise for the development of more effective and personalized treatments for mood disorders, with the potential to significantly improve clinical outcomes. While the findings discussed in this Research Topic offer promising directions, it is essential to emphasize that many of the identified alterations should be considered potential biomarkers. Further validation in larger, longitudinal, and translational studies is warranted to ascertain their clinical applicability and reliability. Such scientific prudence remains vital to advancing the field responsibly.

## References

[1] Drevets WC. Neuroimaging studies of mood disorders. Biol Psychiatry. (2000) 48:813–29. doi: 10.1016/S0006-3223(00)01020-9, PMID: 11063977

[2] Konarski JZ Mcintyre RS Soczynska JK Kennedy SH. Neuroimaging approaches in mood disorders: technique and clinical implications. Ann Clin Psychiatry. (2007) 19:265–77. doi: 10.3109/10401230701653435, PMID: 18058284

[3] Kandilarova S Najar D Velkov N Stoyanova D Zlateva G Todorova A-A. Neuroimaging aspects of interception in mood disorders: A systematic review. J Affect Disord. (2025) 368:686–94. doi: 10.1016/j.jad.2024.09.125, PMID: 39303889

[4] Al-Sharif NB Zavaliangos-Petropulu A Narr KL. A review of diffusion MRI in mood disorders: mechanisms and predictors of treatment response. Neuropsychopharmacol. (2025) 50:211–29. doi: 10.1038/s41386-024-01894-3, PMID: 38902355 PMC11525636

[5] McQuaid RJ. Transdiagnostic biomarker approaches to mental health disorders: Consideration of symptom complexity, comorbidity and context. Brain Behavior Immun - Health. (2021) 16:100303. doi: 10.1016/j.bbih.2021.100303, PMID: 34589795 PMC8474161

[6] Watanabe K Jogia J Yoshimura R. Editorial: Recent developments in neuroimaging in mood disorders. Front Psychiatry. (2024) 15:1371347. doi: 10.3389/fpsyt.2024.1371347, PMID: 38487582 PMC10938263

[7] Ewen JB Potter WZ Sweeney JA. Biomarkers and neurobehavioral diagnosis. Biomarkers Neuropsychiatry. (2021) 4:100029. doi: 10.1016/j.bionps.2020.100029, PMID: 34430906 PMC8382261

[8] Demeter DV Greene DJ. The promise of precision functional mapping for neuroimaging in psychiatry. Neuropsychopharmacol. (2025) 50:16–28. doi: 10.1038/s41386-024-01941-z, PMID: 39085426 PMC11526039

[9] Xia F Kheirbek MA. Circuit-based biomarkers for mood and anxiety disorders. Trends Neurosci. (2020) 43:902–15. doi: 10.1016/j.tins.2020.08.004, PMID: 32917408 PMC7606349

[10] Natale A Mineo L Fusar-Poli L Aguglia A Rodolico A Tusconi M. Mixed depression: A mini-review to guide clinical practice and future research developments. Brain Sci. (2022) 12:92. doi: 10.3390/brainsci12010092, PMID: 35053835 PMC8773514

[11] Mineo L Rodolico A Concerto C Natale A Pennisi M Tusconi M. Mixed depression: A survey on psychopathological, diagnostic, and therapeutic approaches among a sample of italian psychiatrists. Clin Pract Epidemiol Ment Health. (2021) 17:331–341. doi: 10.2174/1745017902117010331

[12] Williams LM Whitfield Gabrieli S. Neuroimaging for precision medicine in psychiatry. Neuropsychopharmacol. (2025) 50:246–57. doi: 10.1038/s41386-024-01917-z, PMID: 39039140 PMC11525658

[13] Bayrak M. Metabolic syndrome, depression, and fibromyalgia syndrome prevalence in patients with irritable bowel syndrome: A case-control study. Medicine. (2020) 99:e20577. doi: 10.1097/MD.0000000000020577, PMID: 32502027 PMC7306332

[14] Tang L Tang R Zheng J Zhao P Zhu R Tang Y. Dissecting biological heterogeneity in major depressive disorder based on neuroimaging subtypes with multi-omics data. Transl Psychiatry. (2025) 15:72. doi: 10.1038/s41398-025-03286-7, PMID: 40032862 PMC11876359

[15] Fales CL Barch DM Rundle MM Mintun MA Mathews J Snyder AZ. Antidepressant treatment normalizes hypoactivity in dorsolateral prefrontal cortex during emotional interference processing in major depression. J Affect Disord. (2009) 112:206–11. doi: 10.1016/j.jad.2008.04.027, PMID: 18559283 PMC2825146

[16] Carta MG Fornaro M Primavera D Nardi AE Karam E. Dysregulation of mood, energy, and social rhythms syndrome (DYMERS): A working hypothesis. J Public Health Res. (2024) 13:22799036241248022. doi: 10.1177/22799036241248022, PMID: 38680762 PMC11047225

[17] Yang L Wang Q Yang R Chen B Guo L. Neuroimaging biomarkers in postpartum depression: A comprehensive review of structural, functional, and metabolic alterations. Behav Brain Res. (2025), 115810. doi: 10.1016/j.bbr.2025.115810, PMID: 40925498

[18] Li B Zhang C Chen W Xie G Liang J. Amygdala volume abnormalities and cognitive impairment in major depressive disorder and bipolar disorder II. BMC Psychiatry. (2025) 25:839. doi: 10.1186/s12888-025-07313-1, PMID: 40877858 PMC12395685

[19] Ren J Ma L Wu W Qiu J Zhang Z Hong Y. Activation likelihood estimation meta-analysis of the effects of cognitive behavioral therapy on brain activation in the treatment of depression and anxiety disorders. Depression Anxiety. (2025) 2025:3557367. doi: 10.1155/da/3557367, PMID: 40556954 PMC12187445

[20] Crowther A Smoski MJ Minkel J Moore T Gibbs D Petty C. Resting-state connectivity predictors of response to psychotherapy in major depressive disorder. Neuropsychopharmacol. (2015) 40:1659–73. doi: 10.1038/npp.2015.12, PMID: 25578796 PMC4915248

[21] Kandilarova S Stoyanov D Sirakov N Maes M Specht K. Reduced grey matter volume in frontal and temporal areas in depression: contributions from voxel-based morphometry study. Acta Neuropsychiatrica. (2019) 31:252–7. doi: 10.1017/neu.2019.20, PMID: 31234950

[22] Wang Y Chen L Wang C Yan C Xie G Yang X. Changed ventral striatum structural covariance and grey matter volume in depression during a one-year follow-up. Psychiatry Research: Neuroimaging. (2024) 344:111887. doi: 10.1016/j.pscychresns.2024.111887, PMID: 39236484

[23] Nosari G Delvecchio G Diwadkar VA Brambilla P. Brain imaging in psychiatry. In: Tasman A Riba MB Alarcón RD Alfonso CA Kanba S Ndetei DM Ng CH Schulze TG Lecic-Tosevski D, editors. Tasman’s psychiatry. Springer International Publishing, Cham (2020). p. 1–32. doi: 10.1007/978-3-030-42825-9_115-1

[24] Malhi GS Lagopoulos J. Making sense of neuroimaging in psychiatry. Acta Psychiatrica Scandinavica. (2008) 117:100–17. doi: 10.1111/j.1600-0447.2007.01111.x, PMID: 18028255

[25] Sun L Wang P Zheng Y Wang J Wang J Xue S-W. Dissecting heterogeneity in major depressive disorder via normative model-driven subtyping of functional brain networks. J Affect Disord. (2025) 377:1–13. doi: 10.1016/j.jad.2025.02.033, PMID: 39978475

[26] Zhou N Yuan Z Zhou H Lyu D Wang F Wang M. Using dynamic graph convolutional network to identify individuals with major depression disorder. J Affect Disord. (2025) 371:188–95. doi: 10.1016/j.jad.2024.11.035, PMID: 39566747

[27] Mansoor M Ansari K. Advancing early detection of major depressive disorder using multisite functional magnetic resonance imaging data: comparative analysis of AI models. JMIRx Med. (2025) 6:e65417. doi: 10.2196/65417, PMID: 40663721 PMC12282933

[28] Park E-H Jung MH. The impact of major depressive disorder on adaptive function: A retrospective observational study. Medicine. (2019) 98:e18515. doi: 10.1097/MD.0000000000018515, PMID: 31876742 PMC6946334

[29] Ouali U Aissa A Rejaibi S Zoghlami N Larnaout A Zgueb Y. Hyperactivity and risk for dysregulation of mood, energy, and social rhythms syndrome (DYMERS): standardization of a simple one-item screener versus the mood disorder questionnaire (MDQ). J Clin Med. (2024) 13:4433. doi: 10.3390/jcm13154433, PMID: 39124700 PMC11312798

[30] Congiu P Carta MG Perra A Cantone E Lorrai S Pintus E. Prevalence and risk by age and sex of sleep dysregulation and depressive episodes in bipolar and depressive disorders in a community survey in sardinia, Italy. J Clin Med. (2024) 13:4870. doi: 10.3390/jcm13164870, PMID: 39201012 PMC11355541

[31] Steinmann LA Dohm K Goltermann J Richter M Enneking V Lippitz M. Understanding the neurobiological basis of anhedonia in major depressive disorder — evidence for reduced neural activation during reward and loss processing. J Psychiatry Neurosci. (2022) 47:E284–92. doi: 10.1503/jpn.210180, PMID: 35948341 PMC9377543

[32] Ji X Yin X Yang J Song J He Y Liang S. Early neuroimaging features of subthreshold depression: Abnormalities in frontoparietal white matter microstructure and functional activity. Asian J Psychiatry. (2025) 104:104389. doi: 10.1016/j.ajp.2025.104389, PMID: 39933305

[33] Yang R Daigle BJ Rampersaud R Schultebraucks K. Editorial: Systems biology approaches to psychiatric and psychological disorders: unraveling the complexities. Front Genet. (2025) 16:1547943. doi: 10.3389/fgene.2025.1547943, PMID: 39935834 PMC11810898

[34] Sheline YI Thase ME Hembree EA Balderston NL Nitchie FJ Batzdorf AS. Neuroimaging changes in major depression with brief computer-assisted cognitive behavioral therapy compared to waitlist. Mol Psychiatry. (2025) 30:3579–87. doi: 10.1038/s41380-025-02945-x, PMID: 40069356 PMC12240849

